# Pathophysiological and molecular insights into allergic conjunctivitis: an expanding analysis of immunological endotypes

**DOI:** 10.3389/falgy.2026.1863898

**Published:** 2026-06-23

**Authors:** Henry Velazquez-Soto, Lorenzo Islas-Vazquez, Marisa Cruz-Aguilar, Estefanía Castillo-Balcazar, Ana X. Yhmoff-Sanchez, María C. Jiménez-Martínez

**Affiliations:** 1 Department of Immunology, Research Unit, Institute of Ophthalmology Conde de Valenciana Foundation, Mexico City, Mexico; 2 Department of Biochemistry, Faculty of Medicine, National Autonomous University of Mexico, Mexico City, Mexico

**Keywords:** allergic conjunctivitis, endotypes, epigenetics, genetics, immunological response, miRNAs, tear cytokines

## Abstract

Allergic conjunctivitis (AC) is an ocular surface disease characterized by immune-mediated inflammation that can significantly impair quality of life. This review explores recent advances in understanding the immunological and molecular mechanisms underlying AC, highlighting local and systemic immune responses and their roles in disease pathogenesis and progression. Particular attention is given to the influence of genetic and epigenetic factors, which may contribute to individual susceptibility and variability in clinical presentation. Finally, this review discusses emerging evidence on clinical phenotypes and immunological endotypes in ocular allergy, emphasizing their relevance for the development of targeted, personalized, and precision therapies.

## 1 Introduction

Allergic conjunctivitis (AC) is an inflammatory process of the ocular surface of the eye affecting the conjunctiva, lid, cornea, lachrymal gland, and even the tear film (1). AC is characterized by redness, erythema, periocular swelling, chemosis, itching, pruritus, and tearing (2). More than a single disease, AC comprises distinct clinical forms. Two acute forms, seasonal (SAC) and perennial allergic conjunctivitis (PAC), and chronic forms, vernal keratoconjunctivitis (VKC), atopic keratoconjunctivitis (AKC), and giant papillary conjunctivitis (GPC). Table 1 presents the relevant clinical characteristics of AC forms and the recognized ocular surface damage mechanisms associated with each (1, 3–17).

**Table 1:** Key ophthalmic findings and immune damage in allergic conjunctivitis^a^.

Feature	Seasonal conjunctivitis	Perennial conjunctivitis	Atopic kerato-conjunctivitis	Vernal Kerato-conjunctivitis	Giant papillary conjunctivitis
Type of Presentation	Acute	Acute	Chronic	Chronic	Chronic
Seasonal Variations	Yes	Sometimes, depending on the antigen	Possible	Yes	No
Age at onset	Mainly Children, but also young adults	Mainly Children, but also Young Adults	Children and Adults with atopic dermatitis^b^	Children and Young Adults	None
Sex predominance	None	None	None	Male: if present under 20 years-old; no differences older than 20 years old	None
Corneal involvement	No	No	Diffuse SPK, corneal nerve abnormalities, shield ulcers.	SPK in some specific areas, shield ulcers, and corneal nerve abnormalities.	Yes
Corneal vascularization	No	No	Sometimes	Rare	No
Limbal Stem Cell Deficiency	No	No	Frequently	Rare	No
Eyelid involvement	No	No	Typical eyelid eczema, meibomian gland dysfunction	Rare	No
Surface Changes	No	No	Tarsal giant papillae, limbal, and fornix changes, rare Trantas dots.	Tarsal cobblestone papillae, Trantas dots, limbal vascularization.	Tarsal giant papillae
IgE sensitization	Yes, mainly aeroallergens	Yes, multiple sensitizations	Yes, multiple sensitizations	Yes, multiple sensitizations	Sometimes
Inflammatory cells at ocular surface	Rare	Sometimes	Mainly Neutrophils	Mainly Eosinophils	Several types of cells, including lymphocytes, neutrophils, and mast cells.

a Contact blepharoconjunctivitis (CBC) is another clinical presentation of allergic conjunctivitis. Therefore, although it could be considered part of the group of allergic eye surface inflammatory diseases, its mechanisms of immune damage differ from those of the main forms of allergic conjunctivitis reviewed here (3, 4). CBC is usually associated with contact dermatitis, and in both cases, sensitized T cells respond to contact antigens and/or haptens, such as ophthalmic drugs, preservatives, cosmetics, or environmental agents, leading to a cell-mediated inflammatory reaction. Thus, CBC contributes to a broader spectrum of ocular and inflammatory skin diseases, but it should be mechanistically differentiated from the mainly IgE-mediated forms of AC.

b Atopic dermatitis is frequently associated with AKC; both diseases share pathogenic mechanisms, including barrier dysfunction, type-2 cytokines, and IgE-related immune responses. Impairment of the skin and ocular surface barriers may facilitate allergen penetration and promote chronic eye inflammation (3, 5, 6).

## 2 Immunological role of the human tear film

The tear film plays a crucial role in maintaining ocular surface health and visual function. It provides multiple protective and physiological functions, including lubrication, prevention of epithelial desiccation, formation of a smooth refractive surface, and supply of oxygen to the avascular cornea. Moreover, it constitutes a primary component of the eye's innate defense system, serving as a barrier against environmental insults and microbial pathogens (18–23). Despite its limited volume (3–10 µL), tear fluid is considered one of the most complex biological secretions in the human body (19, 20, 23). It is composed of water, electrolytes, mucins, lipids, and a wide array of proteins, including enzymes, cytokines, and growth factors, which are secreted through the coordinated action of the lacrimal glands, accessory glands, meibomian glands, and conjunctival goblet cells. The dynamic equilibrium of these components is crucial for maintaining the stability, integrity, and homeostasis of the tear film and the underlying ocular surface epithelia (18–20, 23). Among these structural and protective elements, membrane-associated mucins (MUC1, MUC4, and MUC16) play a central role in epithelial defense. These mucins are enriched at the tips of microplicae, forming a dense and hydrated glycocalyx that provides anti-adhesive and barrier functions at the epithelial-tear film interface (24). In parallel, the secretory mucin MUC5AC, predominantly produced by conjunctival goblet cells, represents one of the most abundant and functionally relevant glycoproteins on the ocular surface, contributing to tear film stability and pathogen clearance (25).

In addition to mucins, the immunoglobulin content of the tear film makes a substantial contribution to immune surveillance. The antibodies IgA, IgG, and IgM are present and help neutralize potentially harmful pathogens and environmental irritants. Notably, IgD is undetectable in human tear fluid, suggesting a limited or absent role in ocular immunity. Among these, secretory IgA (sIgA) is the predominant immunoglobulin in the tear film of healthy individuals, while IgG and IgM are also present in lower concentrations (26–30). Tear antibodies help neutralize a broad spectrum of pathogens that encounter the ocular surface, including bacteria, viruses, and fungi. In addition to immunoglobulins, the tear film contains antimicrobial proteins such as lysozyme, lactoferrin, and lipocalin, which function synergistically to maintain a robust barrier against external insults and preserve ocular surface integrity (18–20, 31–33).

Tear fluid also plays an active role in tissue repair and immunomodulation, as it contains growth factors such as Epidermal Growth Factor (EGF), Transforming Growth Factor (TGF)-β, and Nerve Growth Factor (NGF), which facilitate epithelial cell proliferation, migration, and wound healing (18–20, 23, 32). Disruptions in the production, composition, or distribution of these components have been widely reported in allergy, compromising the stability of the tear film, leading to ocular surface damage.

### 2.1 Soluble mediators in the tear film during ocular allergy

**Figure 1:**
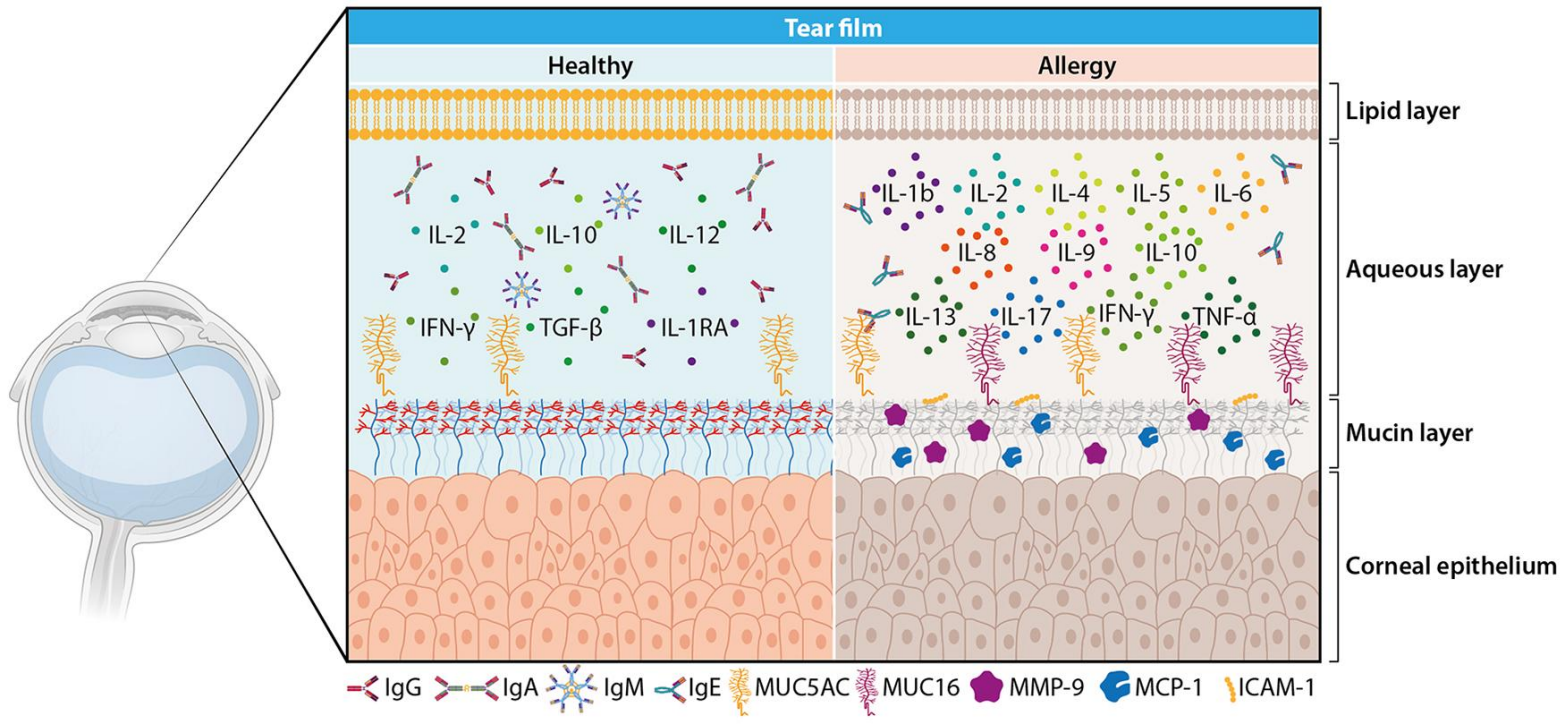
Immunological composition of the tear film in non-allergic eyes compared to those with ocular allergy. The tear film of healthy individuals comprises IL-2, IL-10, IL-12, IFN-γ, TGF-β, and IL-1RA. These cytokines interact with each other and downregulate inflammation at the ocular surface. Protective antibodies, including IgA, IgG, and IgM, provide defense against external pathogens, while MUC5AC ensures the tear film stays adequately hydrated. An increased presence of pro-inflammatory cytokines, including IL-1β, IL-4, IL-5, IL-6, IL-8, IL-9, IL-10, IL-13, IL-17, IFN-γ, and TNF-α, characterizes the tear film in individuals with ocular allergy. Additionally, soluble molecules such as Monocyte Chemoattractant Protein (MCP)-1 and Intercellular Adhesion Molecule (ICAM)-1, as well as enzymes like MMP-9, are prominent. Elevated levels of IgE and MUC16 further contribute to the inflammatory environment observed in ocular allergy.

Numerous soluble molecules have been identified in both healthy and allergic ocular surfaces (Figure 1, and Supplementary Table S1). In acute forms of ocular allergy, the local immune environment is typically dominated by a classical Th2-type proinflammatory profile, characterized by elevated levels of cytokines such as IL-4, IL-5, and IL-13, which promote eosinophil activation, IgE production, and mast cell degranulation. This Th2 profile is often accompanied by increased expression of additional proinflammatory mediators, including IL-1β, IL-6, and Tumor Necrosis Factor (TNF)-α, contributing to the amplification of the inflammatory cascade (21, 26, 30, 34). In contrast, chronic forms of allergic conjunctivitis demonstrate the additional involvement of Th1 and Th17 pathways, as well as a broader and more complex network of soluble mediators, including Interferon (IFN)-γ, IL-17A, TNF-α, and multiple chemokines, proteases, and growth factors (19, 23, 26, 35–37). These shifts in the immune microenvironment prolong inflammation and contribute to epithelial damage, tissue remodeling, and ocular surface dysfunction. Accompanying these immunological changes, alterations in mucin expression have also been documented (38). Notably, patients with chronic ocular allergy exhibit a reduction in MUC5AC, accompanied by compensatory upregulation of membrane-associated mucins, such as MUC1, MUC2, MUC4, and MUC16 (39, 40). These changes may represent an adaptive mechanism to preserve barrier function in the face of persistent inflammation.

The mucin imbalance can also contribute to tear film instability, leading to excess mucous secretion, a clinical feature of severe allergic conjunctivitis. In this inflammatory context, IL-17A stimulates the production of matrix metalloproteinase-9 (MMP-9) (27, 30, 35–37), a proteolytic enzyme implicated in corneal remodeling and the formation of giant papillae (21, 26, 27, 30, 31, 35, 36, 41–43). Such alterations may impact visual function due to increased mechanical stress. The shear forces on the corneal epithelium induce pain, irritation, and surface microtrauma, often preceding the onset of overt clinical symptoms. Additional histological changes, including epithelial cornification and apoptosis, have been observed in advanced cases, contributing to ocular discomfort and fluctuating visual acuity (41).

Adding to this complex immunopathology, elevated levels of total IgE (_T_IgE) have been consistently reported in tears from patients with allergic conjunctivitis (18, 19, 23, 31). It has been recognized that upon recognition of the relevant allergen, IgE bound to mast cells triggers their degranulation, leading to the release of both preformed and newly synthesized inflammatory mediators (44). Total tear IgE has been detected in up to 80% of allergic conjunctivitis cases, with higher concentrations observed in acute forms compared to chronic presentations (29, 45, 46). However, specific IgE (_S_IgE) in tears remains insufficiently characterized. This contrasts with other allergic diseases, where the index _S_IgE/_T_IgE has shown a strong correlation with clinical manifestations (47–50).

### 2.2 Tear cytokine network and clinical phenotype in ocular allergy

A comprehensive analysis of tear cytokine concentrations across the clinical phenotypes of allergic conjunctivitis reveals distinct immunological patterns that may serve as diagnostic or therapeutic biomarkers and provide fundamental insights into the complex pathophysiology of the disease. (See Table 2).

**Table 2:** Tear-soluble mediators in allergic conjunctivitis.

Soluble Molecule	SAC	PAC	AKC	VKC	GPC
Cytokines	IL-1β, IL-2, IL-4, IL-5, IL-6, IL-9, IL-10, IL-12p40, IL-12p70, IL-13, IL-17, IL-17A, IL-17E, IL-17F, IL-21, IL-22, IFN-γ, and TNF-α, Periostin	IL-1β, IL-2, IL-4, IL-5, IL-6, IL-9, IL-10, IL-12p40, IL-12p70, IL-13, IL-17, IL-17A, IL-17E, IL-17F, IL-21, IL-22, IL-23, IFN-γ, and TNF-α.	IL-2, IL-4, IL-5, sIL-6R, IL-13, IL-16, IFN-γ, Periostin	IL-1β, IL-2, sIL-2R, IL-3, IL-4, IL-5, IL-6, sIL-6R, IL-9, IL-13, IL-16, IL-17, IL-21, IFN-γ, and TNF-α, Periostin	IL-4, sIL-6R
Chemokines	CXCL8, CCL11, CCL24	CXCL8	CCL17, CCL24	CCL17, CCL24	CXCL8, CL11, CCL18,
Immunoglobulins	IgA, IgE	IgA, IgE	IgA, IgE	IgA, IgE, IgG, IgM	
Growth Factors	TGF-β, TGF-β1, TGF-β2, TGF-β3	TGF-β, TGF-β1, TGF-β2, TGF-β3		VEGF	
Neuropeptides		α-MSH			
Enzymes and Specific Proteins	Tryptase, ECP		ECP, EDN	Tryptase, ECP, EDN, HMGB 1	Tryptase, ECP, EDN
Complement				C3a des-Arg, C3, Factor B	C3a des-Arg, C3, Factor B

According to these data, chronicity in AKC is characterized by elevated levels of eosinophil cationic protein (ECP) (51–56) and eosinophil-derived neurotoxin (EDN) (52), which reflect prominent eosinophilic inflammation. Increased concentrations of CCL24 (eotaxin-2) and CCL17 (TARC) promote eosinophil and Th2 recruitment (43), while the presence of high quantities of interferon gamma (57, 58) and IL-2 (57) indicates chronic tissue activation and Th1 involvement. IL-4, IL-5, and IL-13 (57–59) reflect a continuous Th2 activation that contributes to ocular damage. This mixed Th1/Th2 response could impact the switching of antibody isotypes, since allergen-specific IgA and high concentrations of total IgE have been reported in AKC (54, 60).

In the case of VKC, a mixed Th1/Th2/Th17 response is observed (54, 57, 58, 61–66), but Eosinophil Cationic Protein (ECP) concentrations are often the highest among all conjunctivitis subtypes (51, 52, 67, 68). This leads to a strong eosinophilic reaction, which is associated with increased vascular endothelial growth factor (VEGF) (69), promoting active tissue remodeling and new blood vessel formation. In addition, the Th2 response, mediated by IL-4, IL-5, and IL-13, can be seasonally exacerbated in VKC, where IL-17 also contributes to the local production of proinflammatory cytokines, including TNF-α, IL-1β, and IL-6 (70), thereby causing further local damage to the ocular surface. At the same time, CCL17 and CCL24 (43), induced by Th2 cytokines, are key chemokines involved in the recruitment of eosinophils. Other cytokines reported in tears of VKC patients are IL-16 (43) and IL-21 (71); IL-16 is a cytokine that serves as a chemoattractant for eosinophils, macrophages, and Th2 lymphocytes, stimulating monocytes to secrete pro-inflammatory cytokines (72), and IL-21, on the other hand, is involved in the apoptosis of Forkhead Box P3 (FOXP3)⁺ regulatory T cells in animal models (73) but also plays a regulatory role in B cell function by suppressing IgE class-switching (74). Interestingly, IL-21 has also been shown to induce class-switching toward IgG in both animal models and humans (75). In line with this, allergen-specific IgG and specific IgA have been detected in the tears of VKC patients (76). In parallel, periostin, a matricellular protein implicated in chronic inflammation and tissue remodeling, has also been detected in the tears of patients with AKC and VKC, with notably higher levels reported in AKC (59). Periostin, produced by fibroblasts, epithelial, and endothelial cells in response to IL-4 and IL-13, contributes to the pathogenesis of allergic diseases such as asthma and atopic dermatitis by inducing the expression of proinflammatory cytokines (77). The coexistence of periostin and non-IgE antibody isotypes in tears raises the possibility that chronic allergic inflammation at the ocular surface synergizes both type 2 cytokine-driven tissue remodeling and IL-21-mediated humoral modulation, contributing to disease chronicity and severity.

On the other hand, damage in GPC is mediated by the complement system, as suggested by the detection of Factor B, C3, and C3a-Des-Arg.2 (78). It confirms that GPC is not predominantly Th2-mediated; however, it could coexist in some individuals. Enzymes released by mast cells, including tryptase and ECP, have also been reported (51, 52, 79), albeit in lesser quantities than those found in VKC or AKC. Other proteins, such as High Mobility Group Box 1 (HMGB-1), can increase inflammation by acting as a Damage-Associated Molecular Patterns (DAMP). Relevant chemokines in GCP include CCL18 (80), which acts as an antagonist to CCL11 (eotaxin-1), CCL11 (81), and CXCL8 (80) are also reported in GPC. Interestingly, sIL-6 has been reported in AKC, VKC, and GPC (82) and could play a pivotal role in trans-signaling mechanisms, enabling IL-6 to activate cells that do not express the membrane-bound IL-6 receptor (IL-6Rα), for example, fibroblasts, epithelial cells, and endothelial cells, contributing to chronic inflammation, tissue remodeling, and neovascularization.

In contrast, SAC and PAC exhibit a predominant Th2 profile, characterized by the presence of IL-4, IL-5, IL-6, IL-9, IL-10, and IL-13 (21, 34, 59, 61, 64, 83) but with very low concentrations of IFN-γ, IL-12, ECP and Periostin (21, 51, 52, 59, 83–86) compared to chronic forms. Seasonal conjunctivitis typically resolves on its own and is often associated with exposure to environmental allergens during specific seasons. On the contrary, PAC is associated with low-grade, persistent inflammation, typically against ubiquitous antigens, such as *Der p* or dust mites. The presence of α-melanocyte-stimulating hormone (α-MSH) (87) suggests a neuroimmunoregulatory component. At the same time, modest elevations in IFN-γ (21, 83) when compared with SAC may reflect low-level, chronic, and persistent activation. In seasonal and perennial allergic conjunctivitis, TGF-β and IL-10 likely play an immunoregulatory role at the ocular surface. Although typically expressed at low levels, TGF-β or IL-10 (21, 34, 83) may contribute to the induction of regulatory T cells (Tregs), suppression of Th2-mediated responses, and preservation of epithelial barrier function, particularly in PAC, where chronic antigen exposure necessitates sustained immunomodulation. CXCL8 is a chemokine involved in conjunctival inflammation in both SAC and PAC (21, 34), while CCL11 and CCL24 are mainly reported in SAC (88, 89). These chemokines contribute to the local inflammatory process at the ocular surface, characterized by the recruitment of neutrophils and eosinophils, respectively. IL-17 has also been recently described in both of the acute forms of allergic conjunctivitis (21, 83). In particular, IL-17A and IL-17F activate eosinophils to release CXCL1, CXCL8, IL-1β, and IL-6, favoring local inflammation (90). Moreover, IL-17 may act as an amplifying cytokine in allergic responses triggered or exacerbated by external factors, such as pollutants, as has been demonstrated in animal models of allergic conjunctivitis (91).

In addition to the soluble mediators previously discussed, several studies have identified other molecules associated with ocular allergy, although without specifying the clinical phenotype (Supplementary Table S2). Among them, vasoactive intestinal peptide (VIP), calcitonin gene-related peptide (CGRP), and substance P (SP) have been implicated in ocular allergy since they are increased after the conjunctival provocation test (92), reinforcing the idea of neuroinflammatory processes involved at the ocular surface.

Several other chemokines involved in chronic inflammation, such as CCL3, CCL5, CCL7, CCL23, and CCL25 (93, 94), have been reported in allergic conjunctivitis. CCL3 functions as a chemoattractant for activated lymphocytes and macrophages via CCR1 and CCR5; studies in animal models suggest it is released during mast cell degranulation (95); CCL5 acts on T cells, monocytes, and eosinophils, contributing to local Th1/Th2 inflammation in respiratory allergic diseases through CCR1, CCR3, and CCR5 (96); CCL7 is a pleiotropic chemokine that signals through CCR1, CCR2, and CCR3, thereby attracting monocytes, macrophages, neutrophils, eosinophils, and basophils. All these cells are involved in acute and chronic forms of allergic conjunctivitis. Notably, CCL7 has been identified as a key chemokine in a murine model of allergic conjunctivitis, where it enhances mast cell degranulation (97) CCL23, which signals via CCR1, is produced by eosinophils in response to IL-5, Granulocyte-Macrophage Colony-Stimulating Factor (GM-CSF), or IFN-γ (98) and acts on neutrophils, T cells, and monocytes; it has also been implicated in neovascularization (99). Thus, its role could be more critical in chronic forms of allergic conjunctivitis. Finally, CCL25 mediates its effects through CCR9, a chemokine receptor associated with mucosal homing. Interestingly, elevated frequencies of circulating CD4⁺CCR9⁺ T cells have been reported in PAC patients (100), suggesting a role for mucosal trafficking in its pathogenesis. Nevertheless, more information is needed to relate to clinical phenotypes and tear chemokine crosstalk in human allergic conjunctivitis.

Interleukin-33 (IL-33), an epithelial-derived alarmin, has been detected in the tears of patients with AC (94). Notably, IL-33 can activate type 2 innate lymphoid cells (ILC2s) to produce IL-5 and IL-13, thereby driving the innate inflammatory response or contributing to the allergen-induced inflammation (101). In addition, other soluble proteins, such as promotilin (MLN), pancreatic lipase-related protein 2 (PNLIPRP2), contactin-associated protein-like 2 (CNTNAP2), and integrin alpha 6 (ITGA6) (94), have also been identified in tears from AC patients; however, their pathophysiological roles and clinical relevance remain unclear.

These complex interactions among soluble mediators become even more intricate when considering the coordinated activity of innate and adaptive immune cells. Investigating these cellular subsets in humans poses additional challenges, particularly due to the anatomical and functional constraints of the ocular surface. Direct assessment often requires invasive techniques such as conjunctival biopsies or impression cytology to characterize cellular involvement. Alternative approaches, such as animal models, *in vivo* confocal microscopy, and searching in peripheral blood from patients, have been employed to elucidate the role of immune cells in allergic conjunctivitis.

## 3 Innate immunity in allergic conjunctivitis

It is generally accepted that innate immunity cells are the first responders when facing an antigen. Innate immunity plays a vital role in recognizing foreign substances, such as allergens, at entry sites, conveying signals to adaptive immunity through antigen presentation, costimulatory molecule expression, and the release of soluble mediators, including cytokines and chemokines. In the context of allergic conjunctivitis, the ocular mucosa is in an inflammatory state where innate immunity actively participates in response to an allergen encounter (102). At first, the immune privilege of the eye was thought to stem from the absence of immune cells in the healthy cornea (103). However, subsequent studies demonstrated the presence of resident MHC-positive antigen-presenting cells (APCs) within the corneal tissue (104, 105). Further *in vivo* confocal microscopy studies have shown dendriform cells (Dendritic cells and Langerhans cells) reside in the human cornea and conjunctiva (106).

### 3.1 Dendritic cells

Dendritic cells (DC) are primarily located in the peripheral corneal epithelium and stroma, with their density decreasing toward the central cornea. While their role in other allergic diseases is well established (107), studies specifically addressing dendritic cell involvement in human allergic conjunctivitis remain limited. Nonetheless, available evidence suggests dynamic changes in dendritic cell density in both cornea and conjunctiva. During allergic inflammation, DCs exhibit a mature phenotype, which reverts to a basal state during asymptomatic phases of ocular allergy, with reduced numbers in the conjunctiva but maintaining higher numbers in cornea (108–110). Experimental allergic conjunctivitis (EAC) mouse models have shown mDCs (CD11c + CD11b+) and pDCs (CD11c + mPDCA-1+) increase in the tarsal, fornix, and bulbar regions of the conjunctiva after allergen exposure. In a similar approach, it was demonstrated that Programmed Cell Death-Ligand 2 (PDL2)^+^ cells (presumably DCs due to PDL2 expression restriction) infiltrate the conjunctiva of mice during experimental allergic conjunctivitis (111). In line with these data, biopsies of patients with AKC reveal epithelial changes, goblet cell proliferation, mast cell infiltration, and eosinophil invasion of the epithelium, as well as mononuclear cell infiltration of the substantia propria, accompanied by granuloma formation. This granuloma formation exhibits a higher proportion of dendritic cells compared to controls (112). Studies in patients with VKC have shown increased expression of MHC II, CD86, and CD83 in DCs derived from peripheral blood monocytes; however, these cells exhibit a reduced ability to activate T cells compared to those from healthy donors (113).

### 3.2 Langerhans cell

Langerhans cells (LC) are APCs distinct from origin, phenotype, and function from DCs; LCs are best known for residing in the epidermis and continuously surveying the microenvironment, coordinating a state of continuous tolerance (114, 115). Studies in humans and mice have confirmed that CD207^+^ Langerhans cells can be found in the conjunctiva and central corneal epithelium, and the number of LCs increases under inflammatory conditions (116–118). Immunostaining of conjunctiva biopsies from VKC patients revealed an increased number of infiltrating CD1a^+^ CD86^+^ cells in both the stroma and epithelium (119).

### 3.3 Macrophages

**Figure 2:**
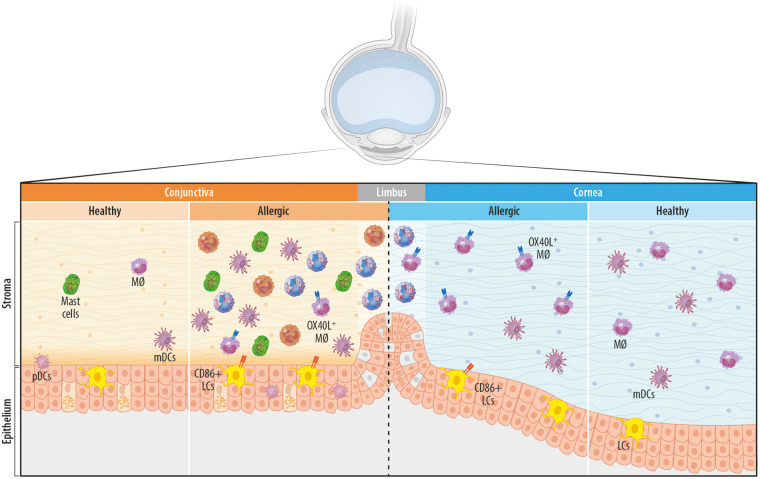
Alterations of innate immune response in allergic conjunctivitis. Studies in animal models and humans have shown that resident innate immune cells are present in the healthy conjunctiva and cornea. During allergic conjunctivitis, mDCs and pDCs increase their number in the bulbar, tarsal, and fornix conjunctiva as well as in the peripheral cornea. CD86^+^ Langerhans cells also increase their number in the conjunctiva of patients. Higher numbers of OX40L^+^M2 macrophages can be found in the conjunctiva in AC. During the symptomatic phase of AC, the number of mast cells, eosinophils, and neutrophils are increased in the conjunctiva.

Distributed in lymphoid and non-lymphoid tissues, macrophages participate in responding to inflammatory stimuli, but also in the clearance of cellular debris, in tissue repair, and in some metabolic functions (120). Macrophages are mainly distributed in the center and peripheral human corneal stroma (121). Macrophages can be broadly classified into M1 and M2 phenotypes according to their activation profile and functional properties. M1 macrophages, also known as classically activated macrophages, are mainly induced by pro-inflammatory stimuli such as IFN-γ and microbial products. They are characterized by the production of inflammatory cytokines, enhanced antigen presentation, and microbicidal activity, contributing to host defense and tissue inflammation. In contrast, M2 macrophages, or alternatively activated macrophages, are generally induced by cytokines such as IL-4 and IL-13. They are associated with anti-inflammatory responses, tissue repair, extracellular matrix remodeling, angiogenesis, and resolution of inflammation (122). Although little has been explored in humans, experimental allergic conjunctivitis (EAC) mouse models have shown that CD68-expressing cells in the conjunctiva are essential APCs for the establishment of ocular allergy (123). Further studies using pollen-induced EAC proved that the influence of thymic stromal lymphopoietic protein (TSLP) alternatively activates conjunctival macrophages, and these M2 macrophages express the costimulatory molecule OX40L and the chemokines TARC (CCL17) and MDC (CCL22) (124). These chemokines predominantly attract Th2 lymphocytes, contributing to allergic inflammation. (Figure 2). Recently, it has been reported that M1 macrophages activated by particulate matter less than 2.5 µm induced TSLP and exacerbated the ocular inflammatory response in a mouse model of allergic conjunctivitis (125).

### 3.4 Granulocytes

Granulocytes are a highly heterogeneous group of cells derived from a myeloid common progenitor in the bone marrow, giving rise to neutrophils, basophils, eosinophils, and mast cells. These cells are characterized by specific granules in their cytoplasm and a high capacity for migration in response to chemoattractant stimuli (126). Seminal works studying granulocytes in conjunctival biopsies demonstrated that mast cells, eosinophils, and a few neutrophils can be found in the healthy lamina propria of the conjunctiva. These mast cells, eosinophils, and neutrophils increase in number in symptomatic SAC patients, and eosinophils increase in the pollen season (127, 128). Since the importance of mast cells in allergic conjunctivitis, further studies have focused on exploring the function of these cells. Through immunostaining of conjunctival biopsies from SAC patients, it was demonstrated that tryptase+-chymase + mast cells are the primary producers of IL-4, IL-5, IL-6, and IL-13 (127). In patients with rhinoconjunctivitis, nasal allergen challenge has been shown to induce dynamic changes in the ocular surface, including a time-dependent increase in eosinophils, neutrophils, and macrophages between 1 and 30 h post-exposure (129), supporting the nasal-ocular inflammator*y* axis seen in clinical contexts (allergic rhinoconjunctivitis). Interestingly, in an EAC model involving sublingual desensitization, eosinophil infiltration at the ocular surface was reduced, correlating with clinical improvement (130). In addition, biopsies from patients with VKC show high infiltration of eosinophils and monocytes/macrophages, both of them expressing VEGF, TGF-b, and Fibroblast Growth Factors (FGF), cytokines involved with tissue remodeling (131).

### 3.5 Innate lymphoid cells (ILCs)

ILCs are a heterogeneous family of immune cells that lack antigen-specific receptors but rapidly respond to environmental and inflammatory signals. They are broadly classified into ILC1, ILC2, and ILC3 subsets, which mirror the functional profiles of Th1, Th2, and Th17/Th22 cells, respectively. ILCs act as early immune effectors that bridge innate and adaptive immunity and contribute to tissue homeostasis and immune-mediated diseases (132, 133). Rag2-deficient mouse models of contact lens-induced conjunctival inflammation have demonstrated that ILC2s can be found in conjunctival and lacrimal gland tissues, and that depletion of ILCs leads to a diminished number of infiltrating eosinophils (134). In a model of mice overexpressing IL-33, which develop spontaneous keratoconjunctivitis, ILC2 were found to be the main source of IL-13 and IL-5 (135, 136), suggesting a role in allergic inflammation.

## 4 Adaptive immunity in allergic conjunctivitis

Beyond the well-established role of Th2 cells in allergic inflammation, evidence indicates that additional adaptive immune cell subsets, including various T and B cell populations, play a crucial role in the pathogenesis of ocular allergy. Previously, we discussed the tear cytokine network and its association with clinical phenotypes. In this section, we present evidence supporting the involvement of specific T cell subsets in the immunopathogenesis of allergic conjunctivitis. This evidence comes from both human studies and experimental models. It is based on identifying T cell participation in conjunctival and corneal tissue, their detection in tear fluid, or their contribution to local allergic dysregulation through systemic immune responses.

### 4.1 Th2/Th1 lymphocytes

Th2 lymphocytes are essential for mediating immune responses against extracellular pathogens, mediating humoral immune responses, and as mentioned above, playing an important role in allergic diseases. Th2 subpopulation is characterized by the production of IL-4, IL-5, and IL-13 (137); while Th1 lymphocytes are involved in the host defense against intracellular pathogens. The production of IL-2 and IFN-γ characterizes this subpopulation. Th1 has also been responsible for the development of certain autoimmune diseases. According to the classical immunological paradigm, Th1 and Th2 responses are mutually regulatory; IFN-γ produced by Th1 cells can suppress Th2-mediated effects by downregulating IL-4 expression, highlighting a dynamic cross-regulatory mechanism between these two subsets (138).

In patients with allergic rhinitis who exhibited conjunctival responses following allergen exposure, lymphocytes were detected at the ocular surface approximately 24 h post-challenge, mirroring the kinetics of IL-4 expression and suggesting a coordinated Th2-mediated immune activation (129). On the other hand, IL-4 knockout (KO) mice exhibit minimal clinical symptoms, less eosinophil infiltration, and a decrease in serum IgE after challenge with allergens (139). In addition, diminishing the Th2 signaling pathway alleviates the clinical symptoms and inflammation in EAC models (130).

Intraepithelial lymphocytes showing Th1/Th2 phenotype have been reported in SAC and PAC patients, with a higher percentage of both cell subtypes in PAC patients (21). Evidence from animal models remains controversial regarding the role of IFN-γ. While some studies suggest a protective effect of IFN-γ in ocular allergy (139), others report that neutralization of IFN-γ during ocular allergen challenge reduces the allergic response (140). This is consistent with the fact that IFN-γ has been associated with the most severe and chronic forms of ocular allergy, such as VKC and AKC, in which levels of IFN-γ correlate with disease severity (58). In this context, our group has investigated the expression of CD30 on T lymphocytes in VKC patients, demonstrating that CD4⁺CD30⁺ T cells are the predominant producers of IL-4, IL-5, and IFN-γ upon allergen *in vitro* stimulation. These findings suggest that this specific subpopulation may represent a key source of both Th1 and Th2 cytokines during the immune response in VKC (141).

### 4.2 Th9 lymphocytes

Th9 lymphocytes are a recognized subset of T-lymphocytes that produce high amounts of IL-9. Although initially thought to be part of the Th2 lineage, differentiating under the influence of TGF-β in Th2-polarized environments, Th9 cells are now recognized to follow a distinct transcriptional and signaling pathway (142, 143). This subpopulation has been studied in conditions such as asthma, atopic dermatitis, and inflammatory bowel disease. Notably, its presence and function have not been sufficiently investigated in allergic conjunctivitis, even though it has been reported to increase in the peripheral blood of patients with rhinoconjunctivitis (144). In line with this data, it has been reported that in local allergic rhinitis, nasal allergen challenge leads to the accumulation of Th9 cells (145), suggesting the involvement of specific allergen Th9 cells in promoting IgE local production by B cells.

### 4.3 Th17/Th22 lymphocytes

Th17 lymphocytes are essential for the immune response against extracellular bacteria and fungi. Still, in pathogenic conditions, they are associated with several autoimmune diseases, including psoriasis, multiple sclerosis, rheumatoid arthritis, and inflammatory bowel disease (146). Th17 cells produce mainly IL-17A, IL-17F, and IL-22 (146, 147). The major function of IL-17 is to recruit and activate neutrophils, whereas IL-22 stimulates cells at the mucosal barrier to produce antimicrobial peptides, proinflammatory cytokines, and chemokines (148).

Contradictory evidence has been reported related to Th17 in ocular allergy; in an IL-17 KO mouse, endogenous IL-17 was insufficient for the development of EAC (149); on the contrary, deficiency of IL-27 induces a strong Th17 response that aggravates Th2 allergic conjunctival inflammation (150). In line with the pathogenic role of Th17 cells to ocular damage, IL-17A and IFN-γ are absent in CCR6KO and CXCR3KO mice; the ablation of either CCR6 or CXCR3 makes mice resistant to developing experimental dry eye disease (151). Conversely, Th17 cells have been reported in healthy human conjunctiva and increased in both SAC and PAC, with a slight predominance in SAC (21). As discussed previously, IL-17 is present in tears mainly in chronic forms; thus, neutrophil infiltration seen in the conjunctiva might be a consequence of Th17 activity (152), as reported in other allergic diseases.

On the other hand, Th22 subpopulations have a protective function through the induction of antimicrobial peptides and play an important role in maintaining the integrity of the epithelial barriers, as well as promote epithelial healing, including the corneal epithelium (153, 154). However, Th22 is also involved in inflammation, autoimmunity, malignancies, and allergic reactions (154). In asthma, the concentration of IL-22 in the serum of patients was higher than that of healthy subjects, and the level of IL-22 correlated with disease severity (155). In addition, in children with allergic rhinitis (AR) and asthma, IL-22 expression was found in PBMC, and a correlation between IL-22 and serum total IgE levels was demonstrated (156). Taken together, these reports suggest a role of this cytokine in early events involved in the development of these allergic diseases. The distribution of IL-22 secreting cells has been reported in conjunctival-associated lymphoid tissues (CALT) from both mice and humans, in which the main population was Th22, and Th22 cells increase in seasonal allergic conjunctivitis (157).

### 4.4 Regulatory T-lymphocytes (treg)

Treg lymphocytes play an important role in the maintenance of homeostasis, comprising thymus-derived Treg or natural Treg (tTreg or nTreg) and peripheral-derived Treg or inducible Treg (pTreg or iTreg). The nTreg development in the thymus occurs from those that have high-affinity interactions with self-peptides during positive selection. Whereas iTreg are peripherally induced in microenvironments with high TGF-β, IL-10 and RA concentrations (158, 159). In addition to nTreg and iTreg, other T regulatory subpopulations are described such Th3, which produce high amounts of TGF-β, and Tr1 that produce IL-10 (158, 159).

**Figure 3:**
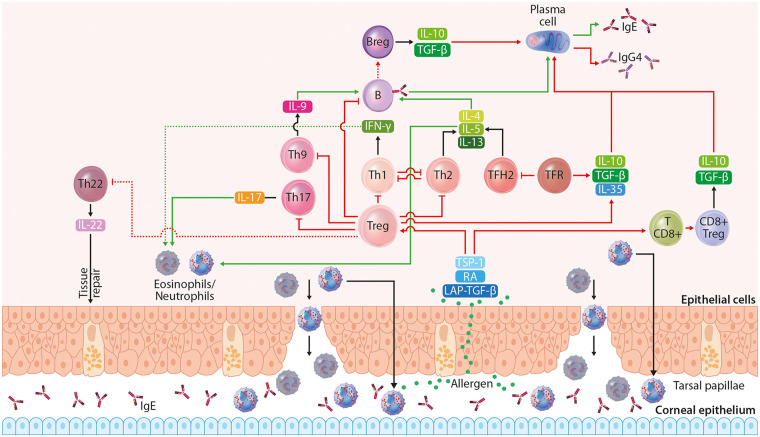
Hypothetical role of T cell subpopulations in ocular allergy. Under normal homeostatic conditions, the ocular epithelium contributes to maintaining immune tolerance by producing immunomodulatory molecules such as thrombospondin-1 (TSP-1), retinoic acid (RA), and the latency-associated peptide of TGF-β (LAP–TGF-β), which collectively generate a local immunosuppressive microenvironment that promotes the differentiation of CD8⁺ regulatory T cells (Tregs). This environment also supports the development of CD4⁺ Tregs, which can suppress the activation and function of multiple T helper subsets, including Th1, Th2, Th9, Th17, and potentially Th22 cells. In contrast, upon allergen exposure, the immune balance shifts toward a proinflammatory response. Th2 lymphocytes become activated and secrete IL-4, IL-5, and IL-13, which drive B cells to produce allergen-specific IgE. Th9 cells may further amplify this Th2-driven response through IL-9 secretion, enhancing IgE synthesis by B lymphocytes and contributing to mast cell activation. Regulatory T cells (Tregs) can counteract this process by inhibiting B cell responses and promoting the differentiation of regulatory B cells (Bregs), which in turn contribute to immune modulation by favoring isotype switching from IgE to IgG4. Additionally, Th1 and Th17 cells have been implicated in both acute and chronic forms of allergic conjunctivitis. These subsets promote neutrophil recruitment, primarily through the production of IFN-γ and IL-17, respectively. Th22 cells, via IL-22 secretion, may play a reparative role by promoting epithelial regeneration during or following allergic inflammation. TSP-1, thrombospondin-1; RA, Retinoic acid; LAP-TGF-β, Latency-associated peptide to TGF-β; TGF-β, Transforming growth factor β; IL-, Interleukin; IgE/G, Immunoglobulin E/G; AC, Allergic conjunctivitis; AKC, Atopic keratoconjunctivitis; VKC, Vernal keratoconjunctivitis; Th, Helper T-lymphocyte; TFH, Follicular helper T-lymphocyte; TFR, Follicular T regulatory lymphocyte; Treg, T regulatory lymphocyte; Breg, B regulatory lymphocyte.

Treg can induce immune tolerance through cell-cell interactions via CTLA-4, PD-1, TIGIT, and LAP (TGF-β) on its cell membrane (158, 160). Also, Treg can suppress proliferation and activation of effector Th1, Th2, Th9, and Th17 lymphocytes through IL-10, TGF-β, and IL-35 (159). Besides, IL-10 can inhibit cytokine production by eosinophil, basophils, mast cells, and APC. In addition, can suppress allergen-specific IgE production, while induce the secretion of IgG4 by B-lymphocytes, as well induce the differentiation of IL-10 Breg lymphocytes (161). (Figure 3).

Therefore, ocular allergy may result from an imbalance between Tregs and pathogenic T cells. A difference in the percentage of Treg and pathogenic T-lymphocytes has been reported in allergic asthma, AR, and dermatitis (162–164). In PAC patients, a high proportion of activated T-lymphocytes after allergen challenge with Der P (*Dermatophagoides pteronyssinus*) (100) was detected, while reduced levels of Treg were shown (165). In addition, the absence of Treg appear to correlate with exacerbation of ocular disease. In allergic mice, the depletion of Treg results in the increase of eosinophil infiltration into the conjunctiva (166, 167), and in allergic and asthmatic patients, the suppressive function of Treg related to FOXP3 expression is reduced compared to the healthy controls (162, 163).

Previously, we reported that in patients with AC, upon allergen stimulation, there is an increase in CCR4 and CCR9 on CD4+ T lymphocytes. Furthermore, increased expression of CD103 and CD108 was detected on helper T cells. These results suggest dysregulation between activated T lymphocytes and Treg lymphocytes, and the increase in markers related to mucous migration might be responsible for the inflammation (100). In addition, we investigated the involvement of TLR4 and alpha-MSH in patients with CA. Our findings suggest that alpha-MSH plays a regulatory role by increasing the percentage of Treg, and a higher proportion of CD4 + TLR4+ T cells was also observed (87).

These studies suggest that ocular allergy may result from impaired Treg activity and that enhancing Treg activity can modulate allergic responses. In this sense, allergen-specific immunotherapy (AIT) has been used as treatment for allergic diseases by increasing allergen-specific Treg and restoring their suppressive capacity to restrain IgE-producing B lymphocytes (168, 169). In particular, in an animal model of ocular allergy, sublingual immunotherapy (SLIT) reduces clinical symptoms and Th2 response (130).

Moreover, the retinal pigmental epithelium (RPE) could induce the differentiation of CD8 + Treg through cell contact via B7-2/Cytotoxic T-Lymphocyte-Associated Protein 4 (CTLA-4) and/or PDL-1 interactions (170). These lymphocytes effectively block the exaggerated immune response, thereby maintaining homeostasis. CD8+ Treg express classical markers of Treg, such as FOXP3, CD25, CTLA-4, and secrete anti-inflammatory cytokines, such as IL-10 and TGF-β, to regulate T-mediated immune responses. Also, CD8+ Treg exert their inhibitory function through CTLA-4-dependent contact to stimulate/polarize tolerogenic antigen-presenting cells (APC) (171, 172).

In asthmatic patients, a decrease in the frequency of CD8 + Treg was reported, and the percentage of CD8 + Treg correlated with the asthma severity (173). On the other hand, an increase in CD8 + Treg after allergen-specific immunotherapy has been observed in patients with allergic rhinitis (174, 175). Therefore, CD8 + Treg cells could cooperate with Treg cells in regulating the allergic immune response.

### 4.5 Follicular helper T-lymphocytes (TFH)

Another subpopulation of T lymphocytes that is essential for the formation of germinal centers (GC), identified as follicular helper T-lymphocytes (TFH), was described in 2009 (176–178). TFH are characterized by the transcription factor B cell lymphoma 6 (Bcl6) and the surface marker CXCR5, a chemokine receptor that promotes B and T cell migration toward follicles. TFH mainly produce IL-21, which is necessary for optimal GC response and plasma cell differentiation (178, 179). Interestingly, TFH has been classified as TFH1, TFH2, TFH17, and T Follicular Regulatory (TFR). TFH2 produce IL-4, IL-5, and IL-13, and promote IgE secretion from B-lymphocytes (180, 181). Whereas TFR co-expresses phenotypical markers of TFH and Treg, and high levels of Treg-associated suppressive molecules that allow it to regulate the response of TFH as well as B-lymphocytes response (177, 180, 182). In allergic diseases, such as AR, atopic dermatitis (AD), and asthma, an increase in TFH, particularly TFH2, has been reported, whereas TFR appears to be reduced (183–186). However, the role of TFH in ocular allergy remains unclear, and fewer studies have focused on TFH cells. In one of them, a mouse model of AC with M-cell deficiency showed reduced GC reaction and less TFH activation after allergen challenge, suggesting that the allergen-specific response depends on close collaboration between these two cells (187).

### 4.6 B lymphocytes

B lymphocytes can produce and secrete a broad range of Ig molecules, depending on the microenvironment. In allergic diseases, the predominant Ig is IgE. Recently, a subset of B-lymphocytes with the capacity to secrete high amounts of IL-10 and/or TGF-β has been reported and defined as Breg lymphocytes (188–190). These Breg lymphocytes have been associated with immune tolerance, with Br1 or Breg10 as the predominant subpopulation (188). Breg acts earlier, facilitating the recruitment of Treg lymphocytes, and plays an important role in autoimmunity and allergy (161). Reduced IL-10 expression has been reported in regulatory B cells, identified as CD19⁺CD38⁺ Breg cells, from patients with perennial allergic conjunctivitis (PAC) (34) decreased number of Bregs might exacerbate late-phase inflammation in allergic conjunctivitis.

The extensive network of cellular communications, cytokine signaling, and tissue-specific responses suggests that local immune behavior is influenced not just by allergens but also by molecular programming. Understanding the genetic and epigenetic basis of these reactions provides insights into how inherited predisposition and environmental variables interact to cause ocular allergic inflammation.

## 5 Genetic and molecular findings in allergic conjunctivitis

Allergic diseases are complex diseases where genetic factors and specific epigenetic changes combined with environmental factors direct the pathological mechanisms of the diseases (191–193). Genetic association studies through linkage and gene mapping have identified hundreds of potential candidate genes associated with allergic diseases, and more recently, the attention has been focused on the contribution of specific epigenetic changes caused by environmental exposure (194).

Heritable atopy studies tested in animal models showed a possible association of the class II MHC gene with the allergic response (195). In humans, there is no complete agreement that HLA class II genes determine immune responsiveness to all allergens; however, they remain as candidates for atopy genes (195–197). While some studies indicated that the HLA-DR4 and DR7 alleles confer susceptibility to asthma by different traditional peptide-binding mechanisms (197, 198), other studies conducted in different locations and with various populations failed to show any evidence of association between HLA class II and the immune responsiveness to the same allergens tested (199). These discrepancies were likely due either to differences in the phenotyping of the diseases or ethnic differences that influence the mechanism of genetic predisposition (200).

The human genetic locus of IgE receptor beta subunit (FcεRI-β) assigned to chromosome 11q has been the subject of many studies as a possible atopy locus, although the results remain controversial. A variant (Ile181Leu) inherited through the maternal line was identified within the fourth transmembrane domain of FcεRI-β of 155 sibling pairs and in a random patient population, which showed a significant association with asthma and rhinitis (201, 202). Attempts to replicate the linkage in a Japanese population or Italian families did not find substantial evidence of maternal allele sharing and the association between the Ile181Leu polymorphism at FcεRI-β and atopy, suggesting that the polymorphism occurs at a lower frequency and that it may be associated with atopy in very restricted populations (203, 204). Notably, no studies to date have specifically explored the role of FcεRI-β polymorphisms in allergic conjunctivitis, highlighting a critical gap in the genetic understanding of this ocular disease. Therefore, further investigation into the association between chromosome 11q and allergic phenotypes, particularly ocular allergy, is warranted, employing updated molecular and genomic tools to improve resolution and population-specific insights.

The observation that IL-4 promotes the Th2 profile and IgE synthesis in B cells, led to the suggestion that the atopy condition could be related to an increase in the signal transduction of its receptor. Sequencing analysis of IL-4 receptor α chain gene from allergic individuals identified an allelic form of the receptor containing an amino acid substitution at position 576 (R576Q) which could affect the interaction of the cytoplasmic domain of the IL-4 receptor with other molecules in signal transduction (205, 206). But the results vary between different populations of allergic individuals.

**Figure 4:**
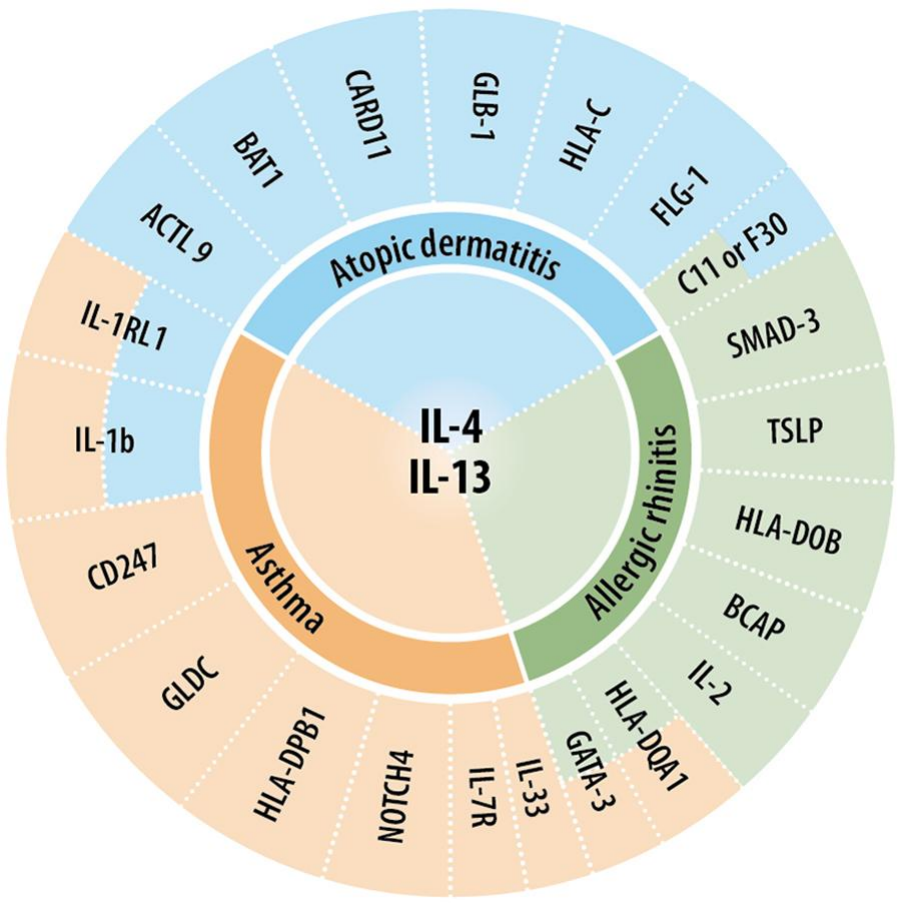
Genomic risk loci in allergic diseases. Relevant genomic risk loci reported in different allergic diseases by a genome-wide association study (GWAS).

The most extensive genome-wide association study (GWAS) has identified about 50 significant risk loci of allergic rhinitis, several with overlapping in asthma such as: IL-4, GATA3, TSLP, IL-13, HLA-DOB, HLA-DQA1, C11orf30, IL-1R, SMAD3, TLR1, TLR10, IL-7R, IL-2, IL-21, and TNFRSF6B, many associated with inflammatory processes and cytokines or molecules of the immune response of Th2 cells (207). (Figure 4).

Currently, the application of next-generation sequencing technology (NGS) in allergic diseases has made it possible to determine the genetic expression profile of the entire genome, or transcriptomics. For example, using RNA-seq, it has been shown that children with asthma have an altered nasal airway transcriptome compared to healthy controls. Analysis of gene expression profiles of allergic rhinitis-derived nasal fibroblasts by RNA-seq highlighted one of the transcription factors, BARX1, whose target genes include IL-33 and IL-6, and which are increased in patients with rhinitis (208).

Despite progress in genomic and transcriptomic research, the genetic basis of allergic conjunctivitis remains poorly defined, with limited studies addressing its specific molecular mechanisms. This gap highlights the need to explore additional layers of gene regulation, particularly epigenetic modifications of non-coding RNAs. Among them, microRNAs (miRNAs) have emerged as critical post-transcriptional regulators that may link genetic predisposition to immune dysregulation in ocular allergy.

### 5.1 miRNAs in allergic conjunctivitis

Transcriptomics has also been extended to the field of microRNAs, whose abnormal expression influences Th1/Th2 polarization and Treg cell/Th17 cell imbalance, promoting inflammation and activation of immune cells (209). At least 200 deregulated miRNAs have been identified by microarray analysis from the bronchial epithelial tissue of asthmatic patients compared with healthy control subjects. Among the miRNAs differentially expressed, notable was the presence of groups of miRNAs with high sequence similarity that derive from distinct DNA sequences, such as members of the miR-34/449 family, which affect NOTCH1 expression and the modulation of T cell-mediated immune responses. However, no clear relationship between miRNA expression and serum IgE level was observed (209).

Similarly, exploration of microRNA profiles in patients with allergic rhinitis determined that the expression of miR-126-5p, miR-19a-5p, and miR-26a-5p was upregulated in contrast with controls and were positively correlated with the severity of disease (210). Alterations in the expression of miRNAs are also responsible for conjunctival inflammation, infection, and blindness. In mouse models, deregulation of miR-146a has been reported to influence the nuclear factor-κB (NF-κB) signaling pathway and the inhibitory effect of regulatory T cells on conventional T cells (211). Furthermore, downregulation of miR-146a induces increased levels of TSLP and its downstream molecules, which increases the total number of inflammatory cells and eosinophils in allergic conjunctivitis (212). Additionally, downregulation of miR-19 increases the levels of TSLP and the phosphorylated signal transducer and activator of transcription 3 (STAT3), as well as other microRNAs (213). Table 3 shows dysregulated miRNAs in different allergic diseases and their functional role in each disease.

**Table 3:** Role of differentially expressed miRNAs in allergic diseases.

**Disease**	**miRNA**	**Expression level**	**Function**	**Type of model**	**Reference**
AR	miR-19a	upregulation	Decrease allergen-induced suppression of IL-10	Human	(232)
AC	miR-19b	downregulation	Inhibition STAT3 signaling via TSLP	Murine	(213)
Asthma	miR-126	upregulation	Affecting IL-4 expression	Human	(233)
AR	miR-135	upregulation	Increase IgE of nasal mucosa, Decreases the expression of IL-4	Murine	(234)
Asthma and AR	miR-143	downregulation	Regulate memory T-cell differentiation	Human	(235)
AC	miR-146	downregulation	Increase TSLP levels, Regulates CD4 ^+^ CD25^−^T cells	Murine	(211, 212)
Asthma and AC	miR-155	upregulation	Regulates proliferation and differentiation of Treg cells	Murine	(236)
AR	miR-187	upregulation	Modulates T-cell response	Human	(235)
Asthma and AR	miR-206	upregulation	Regulation of VEGF pathway	Human	(237)
Asthma and AR	miR-498	upregulation	Suppressing Th17 cell differentiation	Human	(235)
Asthma	Let-7e	downregulation	Modulating Th2 inflammation	Human	(237)

Research on miRNAs in allergic conjunctivitis remains restricted to animal models. This limited evidence underscores the need to expand miRNA research in patients with allergic conjunctivitis, particularly through integrative approaches linking epigenetic modifications and immune pathways (211–213, 232–237).

### 5.2 Epigenetics in allergic conjunctivitis

Epigenetics also plays a key role in both pathological and immunomodulatory conditions of allergic diseases. Recent evidence strongly suggests that allergens stimulate increased expression of histone deacetylase 1 (HDAC1) and contributes to the pathogenesis of allergic rhinitis by increasing pro-inflammatory cytokines (Th2 cytokine levels) and decreasing anti-inflammatory cytokines such as IL-10 (214–216). Studies of allergic rhinitis in patients and nasal epithelial cells have shown that HDAC1 is upregulated in the immune cells compared to healthy controls. Significantly, IL-4 can increase the expression of HDAC1, inducing nasal epithelial barrier dysfunction (217). Similarly, the evaluation of methylated DNA in nasal swabs from pediatric patients by an epigenome-wide association study (EWAS) found multiple differentially methylated regions in genes implicated in allergic asthma, Th2 activation, and eosinophilia (218). Epigenetic changes in asthma have been linked to several exposures to environmental factors such as tobacco smoke, pollutants, viral infections, and the type 2 cytokines (IL-4 and IL-13) during fetal development and in the first years of life. These epigenetic changes are associated with activation of eosinophils and cytotoxic T cells (219–221).

In allergic rhinitis, DNA methylation patterns differentiate allergic patients from healthy people and correlate with increased CD4+ T cells (222). In addition, it has been suggested that sublingual immunotherapy decreases DNA methylation of the Foxp3 gene in memory regulatory T cells, as well as IL-13-promoted thrombospondin-1 gene methylation in B cells (223).

Epigenetic regulation has emerged as a key determinant of immune responses in allergic diseases, involving mechanisms such as histone modifications, DNA methylation, and non-coding RNAs (224, 225). However, to date, no EWAS or targeted analyses of histone modifications have been conducted specifically in allergic conjunctivitis, representing a significant gap in understanding its pathogenic and immunomodulatory mechanisms. Given the clinical heterogeneity of allergic conjunctivitis, integrating epigenetic profiling with immunophenotyping may provide a more profound understanding of disease endotypes.

## 6 Endotypes in ocular allergy

The concept of endotypes has recently been introduced in ocular allergy to improve the understanding of its heterogeneous clinical presentations and underlying immunopathological mechanisms (16). An endotype refers to a disease subtype defined by a distinct functional or biological mechanism, in contrast to phenotypes, which are based solely on observable clinical characteristics (226). In line with advancements in other allergic diseases such as asthma and atopic dermatitis, ocular allergic diseases are now being approached through immunological endotyping. In this context, allergic diseases can be classified according to the dominant immune response profile into Type 2 or non-Type 2. Thus, T2 endotypes are characterized by Th2 cell activation and the production of IL-4, IL-5, and IL-13, leading to IgE synthesis, eosinophilia, and mast cell degranulation. In contrast, an expansion view includes Type 1 (T1) and Type 3 (T3) endotypes, which involve IFN-γ and IL-17-mediated pathways, respectively (16, 226, 227).

This immunological framework provides a valuable lens for interpreting the heterogeneity observed in ocular allergy. Expanding knowledge about allergic conjunctivitis is essential to better understand its complex immunopathology and clinical variability. According to the data reviewed, the ocular allergy endotypes proposed in this work are summarized in Table 4.

**Table 4:** Endotypes in ocular allergy.

Endotype	Clinical Subtype	Immunological Tear-profile mainly reported according to data reviewed	Differential Key Tear-Biomarkers Suggested to Evaluate or Confirm Endotypes	Observations
Classic T2	SAC, PAC	IL-1β, IL-4, IL-5, IL-6, IL-13, TGF-β, elevated IgE, mast cells tryptase + chymase+, eosinophils	IL-4, CCL11, CCL24, CXCL8 Total or specific tear IgE,	Acute allergic inflammatory response, Persistent low-grade inflammation in PAC, probably due to sensitization to ubiquitous allergens (allergen exposome)
Mixed T1/T2	AKC	IL-4, IL-5, IL-13, IFN-γ, IL-2, ECP, EDN, IgE, eosinophils	ECP, IL-4, IFN-γ, CCL17, CCL24, periostin	Chronic inflammation with epithelial damage and tissue remodeling in the palpebral conjunctiva or cornea, Seasonal variations often in VKC
Mixed T2/T1	VKC			
Mixed T1/T2/T3,	VKC	IL-4, IL-5, IL-13, IL-17, IFN-γ, TNF-α, IL-6, IL-21, VEGF, IgE, neutrophils, eosinophils	IL-4, IFN-γ, IL-17, IL-21, CCL17, CCL24, MMP-9, VEGF, ECP, TNF-α, periostin	Severe eosinophilic/neutrophilic inflammation and tissue remodeling, Seasonal variations often in VKC
Highly inflammatory	AKC			
Non-T2 (Complement/Neutrophilic)	GPC	Complement activation (C3, C3a des-Arg, Factor B), neutrophils, mast cells	C3a des-Arg, C3, CCL18	Mechanical or inflammatory trigger
T2-Regulatory-deficient	PAC	Classic T2 endotype plus α-MSH, CGRP, SP, VIP	IL-4, IL-10, TNF-α, TGF-β, α-MSH, CGRP, SP, VIP	Diminished circulating Tregs, or Bregs
T2-Neuro inflammatory	SAC			May coexist with other endotypes?

Furthermore, endotypes are dynamic and can be influenced by various intrinsic and extrinsic factors, including genetic predisposition, differential environmental exposures such as allergens and pollutants (“exposome”), or even microbiome composition. These elements modulate immune responses and contribute to distinct molecular expression profiles in allergic diseases. Such interactions may give rise to subendotypes or so-called “complex endotypes”, characterized by overlapping or evolving immunological features. Understanding this multidimensional complexity in ocular allergy is fundamental, as it may facilitate the integration of precision medicine strategies into the management of allergic eye diseases.

## 7 Perspectives and future directions in ocular allergy research

Ocular allergy, once viewed as a purely Th2-mediated hypersensitivity, is now recognized as a complex immune condition involving coordinated interactions between innate and adaptive immune cells, cytokines, genetic predisposition, and environmental factors. The future of research lies in integrative, mechanism-driven approaches that move beyond descriptive pathology toward personalized understanding. In this context, advancing in the field of ocular allergy requires more systematic documentation of patient-specific variables, including age, sex, allergen exposure, treatment history, symptom severity, and ophthalmological comorbidities, such as dry eye disease (DED) and keratoconus. DED frequently coexists with ocular allergy and shares overlapping symptoms and inflammatory mechanisms, including tear film instability, epithelial barrier disruption, and ocular surface inflammation. Keratoconus has also been associated with ocular allergy, chronic ocular repetitive eye rubbing, and, in some cases, a shared inflammatory microenvironment that contributes to corneal remodeling and disease progression (30, 228–231). In parallel, methodological consistency is needed in tear sampling, sample processing, and the technical methods used in analysis, among other variables. Reinforcing these aspects during research will enhance the ability to correlate clinical phenotypes with the underlying immunological endotypes (T1, T2, T3) and their complex interaction with external factors.

Emerging data in genetics, epigenetics, microRNA, and microbiome in allergic conjunctivitis could be critical for developing or improving treatments, as has been demonstrated in other allergic diseases. Finally, translational models bridging murine data with human clinical phenotypes will help study endotypes and identify candidate biomarkers for personalized interventions focused on immune re-education beyond allergen desensitization, bringing us closer to precision immuno-ophthalmology.
